# The day-to-day influence of trauma exposure and sleep dysfunction on everyday stress in youth at familial high-risk for psychotic disorders

**DOI:** 10.1016/j.schres.2024.10.024

**Published:** 2024-11-24

**Authors:** Elizabeth A. Haudrich, Emily K. Burns, Tina Gupta, Gretchen L. Haas, Leslie E. Horton

**Affiliations:** a Department of Psychiatry, University of Pittsburgh School of Medicine, 3811 O’Hara St, Pittsburgh, PA 15213, United States of America; b Department of Psychology, Ohio University, 22 Richland Ave, Athens, OH 45701, United States of America; c Graduate School of Professional Psychology, University of Denver, 2450 S Vine St, Denver, CO 80210, United States of America; d VISN4 MIRECC at VA Pittsburgh Health Care System, University Drive C, Pittsburgh, PA 15240, United States of America

**Keywords:** Psychotic disorders, Familial high-risk, Sleep, Trauma, Daily stress, Diathesis-stress model

## Abstract

Cumulative research finds that exposure to childhood trauma, sleep dysfunction, and high stress levels are prevalent in youth diagnosed with and at-risk for psychotic disorders. However, few studies have investigated the association between nightly sleep and moment-to-moment stress in youth who are at familial high-risk (FHR) for psychotic disorders with varying levels of exposure to childhood trauma. The current study examined the day-to-day associations between trauma severity, nightly sleep duration, and next-day momentary stress in 19 FHR and 19 non-psychiatric youth (ages 13–19 years, 66 % girls). Ecological Momentary Assessment was used to assess these variables across three longitudinal timepoints (baseline, 6-months, and 12-months). The FHR group reported greater trauma severity and shorter sleep duration than the non-psychiatric group. In the whole sample, trauma severity and reduced sleep duration were associated with next-day momentary stress. While group status did not moderate the association between sleep duration and next-day momentary stress, group status did moderate the positive association between trauma severity and next-day momentary stress, showing that the association was specific to the non-psychiatric group. Lastly, the effect of nightly sleep duration on next-day momentary stress was significant and negative, but only at low levels of trauma severity for the whole sample. Findings offer preliminary insights into the associations between trauma severity, sleep duration, and momentary stress. Furthermore, this design can provide a foundation for future research examining environmental and psychosocial risk factors that contribute to symptom progression and prognosis of youth who are genetically vulnerable to psychosis.

## 1. Introduction

Accumulating research suggests individuals diagnosed with psychotic disorders are more likely to experience childhood trauma (i.e., psychological, physical, emotional or sexual abuse, and emotional neglect before age 17 years; Bernstein et al., 2003) compared to those without the disorder (Üçok and Bıkmaz, 2007; Dennison et al., 2012; Duhig et al., 2015). Therefore, looking earlier on in disease progression among youth at risk for psychosis is of interest as an approach to minimizing adverse outcomes later in life including the emergence of psychopathology. Approximately 86.8 % of individuals at-risk for psychosis have experienced childhood trauma (Kraan et al., 2015a, 2015b); however, research pinpointing its potential impact in at-risk youth is still growing. A recent, nuanced perspective of the traditional diathesis-stress model (Walker and Diforio, 1997) suggests that sleep dysfunction (i.e., disruptions in timing, quantity, or quality of sleep; Locke, 2011) may play a role in the etiology of psychosis – especially among genetically vulnerable and trauma-exposed youth – through its link to psychophysiological stress (Lunsford-Avery and Mittal, 2013). However, the ways in which stress and sleep dysfunction interact prior to the emergence of psychosis are not well understood, especially in vulnerable youth. To investigate these topics, we seek to examine day-to-day associations between childhood trauma, sleep dysfunction, and perceived stress in youth at familial high-risk for psychotic disorders in order to investigate these topics.

Youth who have a first-degree relative with a psychotic disorder are classified as being at familial high-risk (FHR) for psychotic disorders (Maxwell, 1992). The estimated likelihood of developing psychosis for those at FHR is 10–12 % (Gottesman and Erlenmeyer-Kimling, 2001)the importance of identifying possible risk factors for psychosis in order to improve early identification, prevention, and intervention strategies. Research examining psychosis-risk markers often uses the diathesis-stress model as a foundation (Walker and Diforio, 1997), which suggests that stressful events can interact with genetic vulnerability, contributing to the emergence of psychotic disorders. Individuals at FHR and clinical high-risk (CHR) for psychosis (those endorsing attenuated symptoms of psychosis but do not reach the threshold for psychotic disorders; Fusar-Poli et al., 2013), tend to exhibit increased stress as reflected by elevated cortisol levels, self-reported stress, and exposure to stressors (Carol and Mittal, 2015; Walker et al., 2013; Cullen et al., 2020; Brandt et al., 2023; Pruessner et al., 2013). Additionally, higher psychophysiological stress is reciprocally linked to increased psychotic symptoms in at-risk samples (Collip et al., 2011; Cullen et al., 2022).

In the diathesis-stress model, exposure to childhood trauma (referred to as “trauma” throughout) is an important postnatal, environmental stressor in at-risk samples that may be compounded by familial vulnerability. In addition to a 67.1 % prevalence rate of trauma in youth at FHR (Brandt et al., 2022; Berthelot et al., 2022), individuals at both CHR and FHR tend to report more trauma exposure than those only at CHR for psychosis (Santesteban-Echarri et al., 2022; Georgopoulos et al., 2019). This is consistent with reports that trauma appears to predict conversion to psychosis, increased attenuated positive symptoms, and lower everday functioning in psychosis-risk populations (Bechdolf et al., 2010; Loewy et al., 2019; Kraan et al., 2015a, 2015b).

**Fig. 1.**
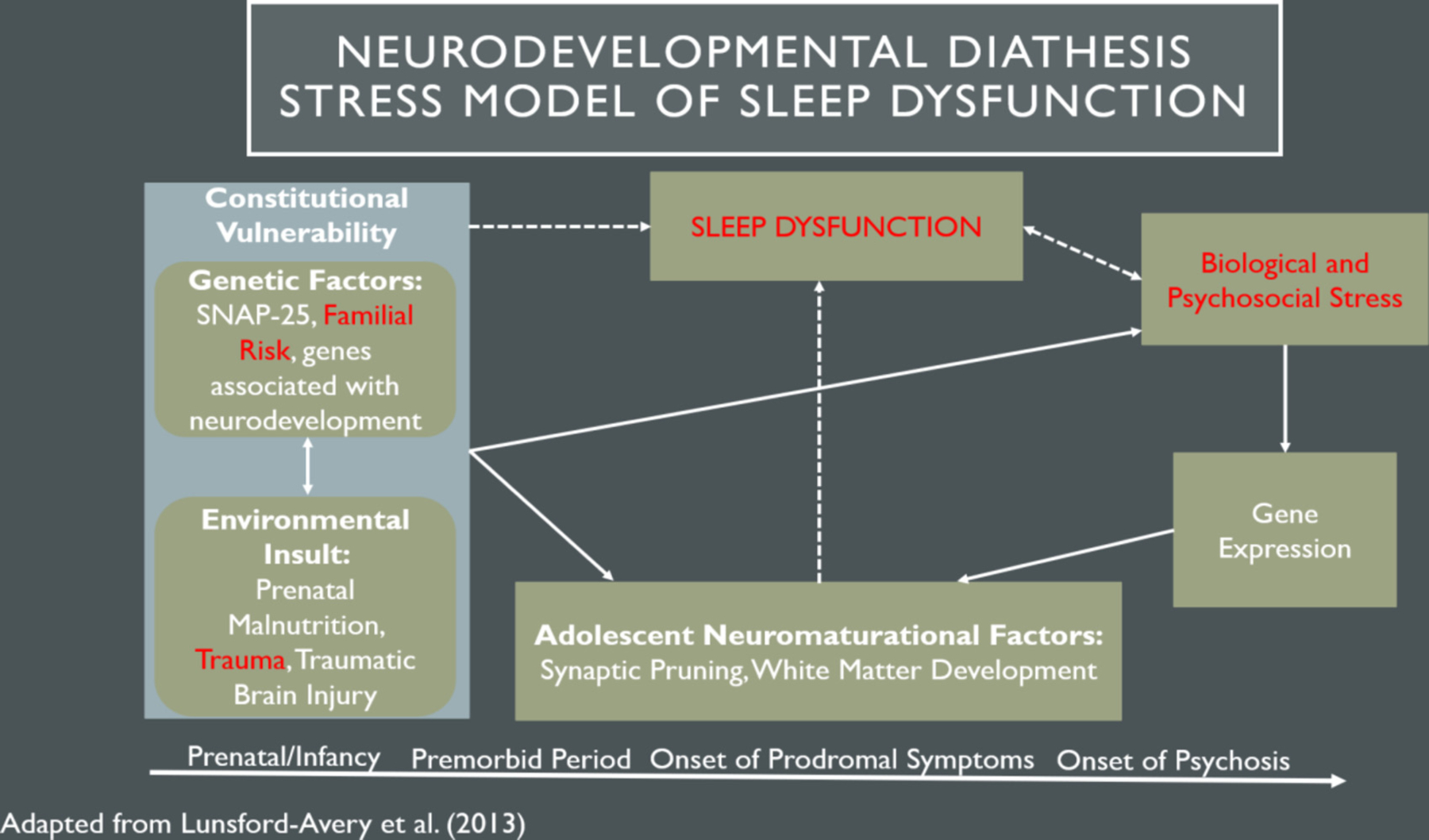
Adapted Neurodevelopmental Diathesis-Stress Model of Sleep Dysfunction *Note*. This figure represents a model adapted from Lunsford-Avery and Mittal (2013) focusing on the role of sleep dysfunction in the Diathesis Stress Model. The bottom line displays the progression from early life to onset of psychosis. The boxes depict variables that may interact to contribute to this progression. The current study examined variables highlighted in red (familial risk, trauma, stress, and sleep). The dotted lines illustrates the hypothesized interactions of sleep in the model. Original Model copyright: © 2013 American Psychological Association. Published by Wiley Periodicals, Inc., on behalf of the American Psychological Association. All rights reserved. For permissions, please email: permissionsuk@wiley.com. (For interpretation of the references to colour in this figure legend, the reader is referred to the web version of this article.)

As mentioned, sleep dysfunction may be a possible factor that interacts with stressful events (e.g., trauma exposure) and genetic vulnerability, further contributing to the emergence of psychosis. Regardless of psychosis-risk or underlying psychopathology, the evolution of sleep dysfunction can be conceptualized as the “perfect storm” (Carskadon, 2011), such that the accumulation of psychosocial/environmental stressors and neurodevelopmental factors throughout childhood contributes to changes and disruptions in sleep patterns later in adolescence. However, sleep dysfunction tends to be one of the first noticeable abnormalities to arise during the prodrome (Yung and McGorry, 1996), with exacerbating effects on symptom progression and psychosis development (Lunsford-Avery et al., 2015; Lunsford-Avery et al., 2017; Zaks et al., 2022). Therefore, from a diathesis-stress perspective, FHR youth may be especially susceptible to the development and consequences of disrupted sleep, as seen in CHR samples (Poe et al., 2017). Expanding upon the traditional diathesis-stress model, Lunsford-Avery and Mittal (2013) propose a Neurodevelopmental Diathesis-Stress conceptualization to explain the role of sleep in the pathophysiology of psychotic disorders (see Fig. 1).

They note that sleep may have an important cyclical interaction with environmental and genetic factors during adolescence. Specifically, environmental and genetic risk factors negatively impact sleep functioning which, in turn, impacts neuromaturation, cognitive function, reactivity to biological/psychosocial stressors, and further alterations in genetic expression. This vicious cycle, particularly during critical developmental periods, may lead to the onset and worsening of psychosis.

However, there is limited research on sleep dysfunction within the diathesis-stress framework for FHR youth. Previous work supports the link between trauma and sleep dysfunction (Andorko et al., 2018; Cardoso et al., 2018; Baddam et al., 2019), with insomnia and trauma-related nightmares being prevalent among trauma-exposed children and individuals with Post-Traumatic Stress Disorder (PTSD; Wamser-Nanney and Chesher, 2018; Milanak et al., 2019). Moreover, emerging evidence suggests an important intersection between trauma, sleep dysfunction, and perceived stress. Not only are trauma and sleep problems independently associated with increased stress levels and exposure (Betz et al., 2021; Beilharz et al., 2019; Yap et al., 2020), Cardoso et al. (2018) also found that trauma is related to sleep disruptions both directly and through increased perceived stress in the general population – a mechanism that may be strengthened by psychosis-risk. To our knowledge, limited work has comprehensively explored this intersection in at-risk individuals despite relatively high rates and associations between trauma, sleep, and stress in psychosis-risk populations – all of which are further implicated in worsening prognosis (Brandt et al., 2023; Lardinois et al., 2011; Laskemoen et al., 2021). Investigating these dynamic relationships in FHR youth may provide support for the diathesis-stress conceptualization of sleep dysfunction as well as insight into how early intervention and preventative programs can target combinations of risk factors in genetically vulnerable youth to improve symptomology and disease prognosis later in life.

### 1.1. Present study

The current study examined the day-to-day interrelationships between trauma, sleep duration, and momentary stress in FHR youth. Based on the literature, it was hypothesized that (H1) FHR youth would report higher trauma severity (e.g., self-report of how traumatic an event was), shorter sleep duration, and higher momentary, perceived stress compared to non-psychiatric control peers; (H2) trauma severity and shorter sleep duration would predict higher next-day momentary stress in the whole sample; and (H3) FHR status would moderate both associations in H2. Lastly, we hypothesized that (H4) in the whole sample, trauma severity would moderate the association between shorter sleep duration and increased next-day momentary stress, such that the link would be the strongest at higher levels of trauma severity.

## 2. Materials and methods

### 2.1. Participants

Nineteen FHR and 19 non-psychiatric control participants (ages 13–19 years) were recruited via community ads and the Pitt + Me Research registry at the University of Pittsburgh, from August 2014 to May 2018, as part of the Adolescent Social Stressors and Thoughts (ASSET) study. Controls were matched for sex at birth and age, as well as ethnicity when possible. This study classified FHR youth as having at least one biological parent that met criteria for schizoaffective or schizophrenia disorder. To assess FHR status, the affected parent completed the Structured Clinical Interview of DSM Diagnosis IV (First, 2005). Additionally, adolescent participants were assessed for current attenuated symptoms of psychosis using the Structured Interview for Psychosis-Risk Symptoms (SIPS; Miller et al., 2002; Miller et al., 2003).

All participants were excluded from the study if they had any of the following: a history of head injury with a loss of consciousness, IQ below 70, unwillingness to abstain from nicotine and caffeine, were unable to understand or speak English at the conversational level, or were taking medications known to influence stress reactivity (e.g., psychostimulants, antipsychotics, or benzodiazepines). FHR candidates with psychotic disorders and non-psychiatric controls with any current mental health diagnoses were excluded.

### 2.2. Procedures and measures

Study procedures were approved by the University of Pittsburgh Institutional Review Board and followed specified guidelines. At three timepoints (baseline, 6- and 12-month follow-up), participants completed structured clinical interviews and three consecutive week-ends of daily self-report questionnaires. Demographic information (e.g., age, sex at birth, race/ethnicity, socioeconomic status (SES)) was provided once at the baseline assessment.

#### 2.2.1. Ecological momentary assessment

Ecological Momentary Assessment (EMA) is a sampling method that repeatedly collects self-reported data of daily thoughts, behavior, and affect in real-time and in participants’ natural environment (Schiffman et al., 2008). The current study uses EMA to examine nightly sleep duration and next-day momentary stress as it minimizes recall bias, maximizes ecological validity, and has been shown to produce reliable data on everyday experiences in the FHR population (Myin-Germeys et al., 2001).

Participants were provided with a smartphone to complete the EMA portion of this study. Using a secure application for WebDataExpress, participants were randomly prompted six times a day between 4 P.M. on Friday to 10 P.M. on Sunday for three consecutive weekends. The questionnaire used was adapted from Silk et al. (2012) for use in the ASSET study. Questionnaires asked about participants’ current experiences including social context, activities, affect, and symptoms of psychosis.

#### 2.2.2. Sleep duration

Sleep duration was assessed by asking participants, “How many hours of actual sleep did you get last night, not including awake time in bed?” on the first EMA prompt of the sampling day. There were 418 sleep data points with more at baseline (*n* = 211) compared to 6-month and 12-month follow-up assessments (*n* = 113 and *n* = 94, respectively).

#### 2.2.3. Trauma severity

Trauma severity was measured using the Childhood Traumatic Events Scale (CTES; Pennebaker and Susman, 1988). Incidence was measured by the 6-item assessment to which participants provided “yes” or “no” responses about experiencing a variety of adverse events (e.g., “Did you experience the death of a very close family member?”). Sixty-three percent of the sample reported at least one traumatic event. Severity was assessed by asking participants to also rate how traumatic these experiences were on a scale from 0 (no exposure) to 7 (extremely traumatic). Trauma severity was an aggregated score across the six trauma domains and was assessed at each timepoint during clinical assessments.

#### 2.2.4. Momentary stress

On each randomly prompted EMA survey, perceived stress was measured by asking participants to rate the statement, “Overall since the LAST beep, how much has stress weighed on you?”, on a scale from 0 (not at all) to 100 (extremely). A total of 2861 EMA stress reports were completed across baseline, 6-month, and 12-month timepoints (*n* = 1376, *n* = 784, *n* = 701, respectively).

## 3. Results

### 3.1. Statistical approach

R version 2023.09.01 (R Core Team, 2023) was used to perform all analyses. Independent *t*-tests, chi-square, and proportion tests were used to assess group differences in demographic, symptom, and compliance/retention measures. Multilevel models (MLMs) were performed to examine group differences and relationships between the primary study variables. Two-level MLMs examined Level 2, between-person predictors (i.e., trauma and sleep duration) and moderators (i.e., trauma and FHR status). Continuous Level 2 predictors were grand-mean-centered, consistent with the recommendations of Cohen et al. (2013) and Luke (2019). Simple slope analyses (Aiken et al., 1991) were used to probe significant two-way interactions between predictor variables.

Additionally, bivariate correlational analyses were conducted to assess associations between the demographic and primary variables. Although momentary stress had significant correlations with participant age, sex at birth, and SES (Hollingshead, 1975) (see supplement Table 1), covarying did not change the magnitude or direction of findings from the primary moderation analysis, β = 0.13, *p* < .01. See supplement for additional analyses of (1) trauma severity and group status predicting sleep duration, and (2) the moderating effects of age on trauma severity and momentary stress.

### 3.2. Descriptive statistics

See Table 1 for descriptive statistics. Overall, there were no significant differences in age, sex at birth, or SES across groups. As expected, FHR youth reported more attenuated positive, negative, disorganized, and general symptoms than non-psychiatric youth. FHR group had more dropouts from baseline to the 6-month assessment and lower EMA response rates relative to the non-psychiatric group. Additionally, three participants (FHR = 1, non-psychiatric = 2) had valid EMA data at baseline and 12 months but missing data at 6 months, and four participants (FHR = 3, non-psychiatric = 1) had low response rates (<30 %). We did not exclude these participants from the primary analyses.

### 3.3. Group differences in trauma, sleep duration, and momentary stress

**Fig. 2.**
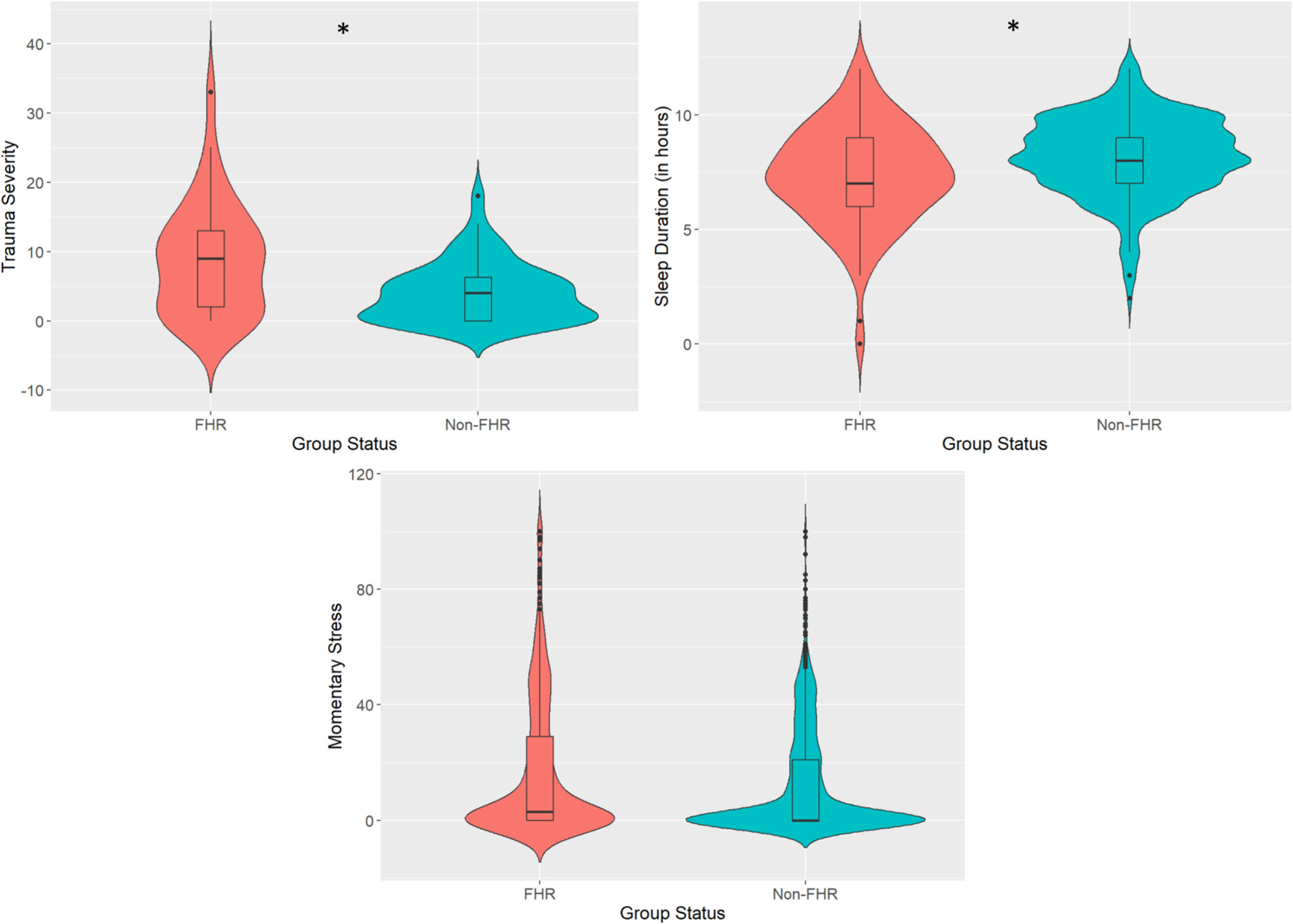
Violin Plots of Group Differences Between Trauma, Sleep, and Momentary Stress Note. p < .05*. Violin plots illustrating the group differences between youth at familial high-risk (FHR) for psychotic disorders and non-FHR peers in trauma severity (left panel), sleep duration (right panel), and momentary stress (bottom panel).

MLMs revealed significant group differences in trauma severity, β = 5.23, SE = 1.96, *p* = .01, and sleep duration, β = −0.81, SE = 0.40, *p* = .05, such that FHR youth reported higher trauma severity and shorter sleep duration than non-psychiatric youth. There was no significant group difference between FHR and control youth for momentary stress, β = 5.49, SE = 4.06, *p* = .18. See Fig. 2 for violin plots of the group differences.

### 3.4. Trauma, sleep, and familial high-risk status predicting momentary stress

See Tables 2, 3 and 4 for results of MLMs examining whether trauma severity, sleep duration, and FHR status predict momentary stress.

**Fig. 3.**
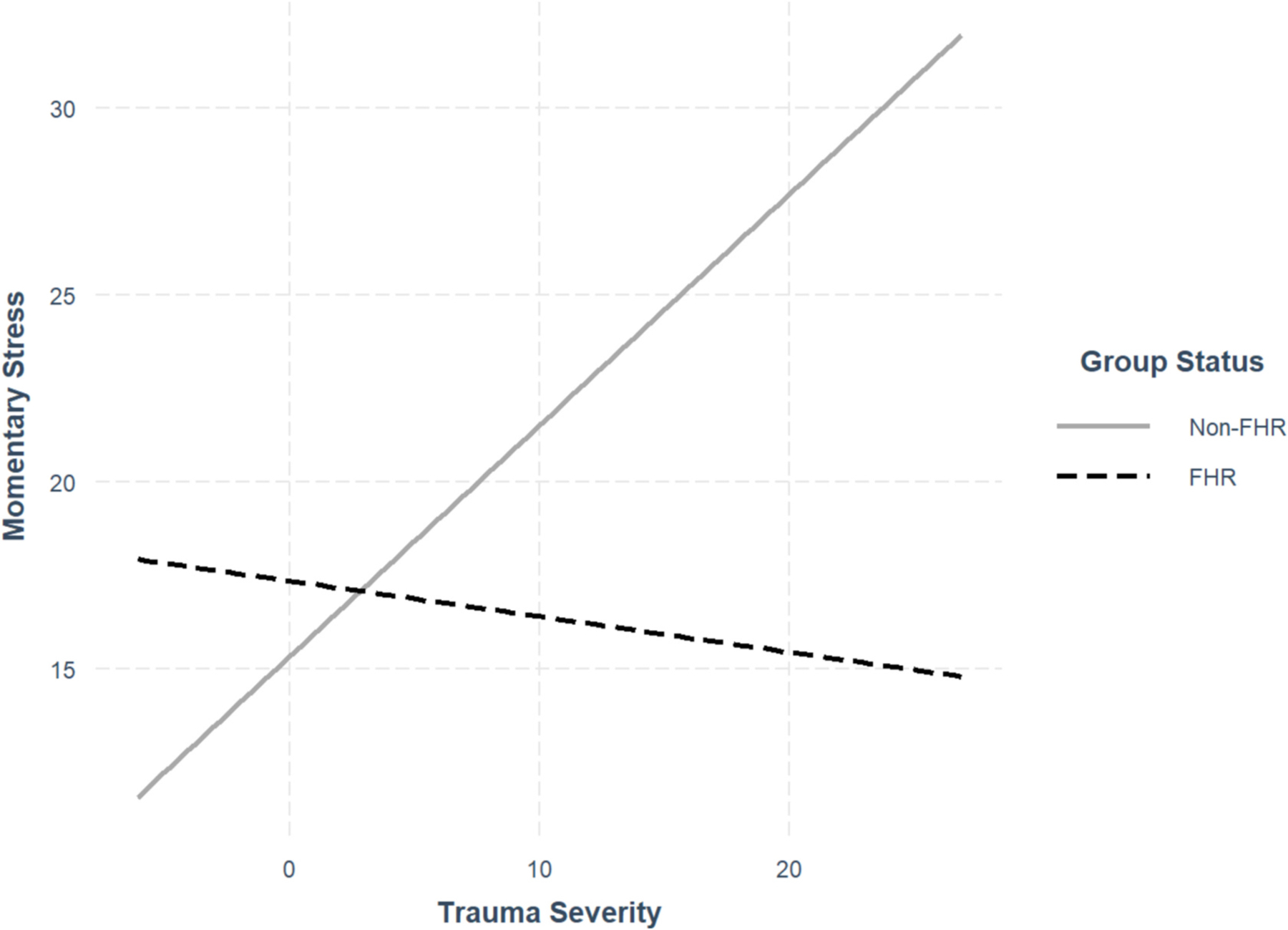
Familial high-risk status moderating association between trauma severity and momentary stress Note. Simple slopes plot illustrates how familial high-risk (FHR) status significantly moderated the positive association between trauma severity and momentary stress such that the association was significant for the non-FHR group but insignificant for the FHR group.

Firstly, higher trauma severity and shorter sleep duration were significantly associated with higher next-day momentary stress in the whole group. Group status did not significantly moderate the association between sleep duration and next-day momentary stress; however, group status did moderate the positive association between trauma severity and momentary stress, such that the association was specific to the non-psychiatric group. Simple slopes analysis supports this result, showing that the association between trauma severity and momentary stress was significant in the non-psychiatric group, β = 0.62, SE = 0.17, *p* < .001, but not significant in the FHR group, β = −0.09, SE = 0.16, *p* = .56 (see Fig. 3 for interaction plot).

**Fig. 4.**
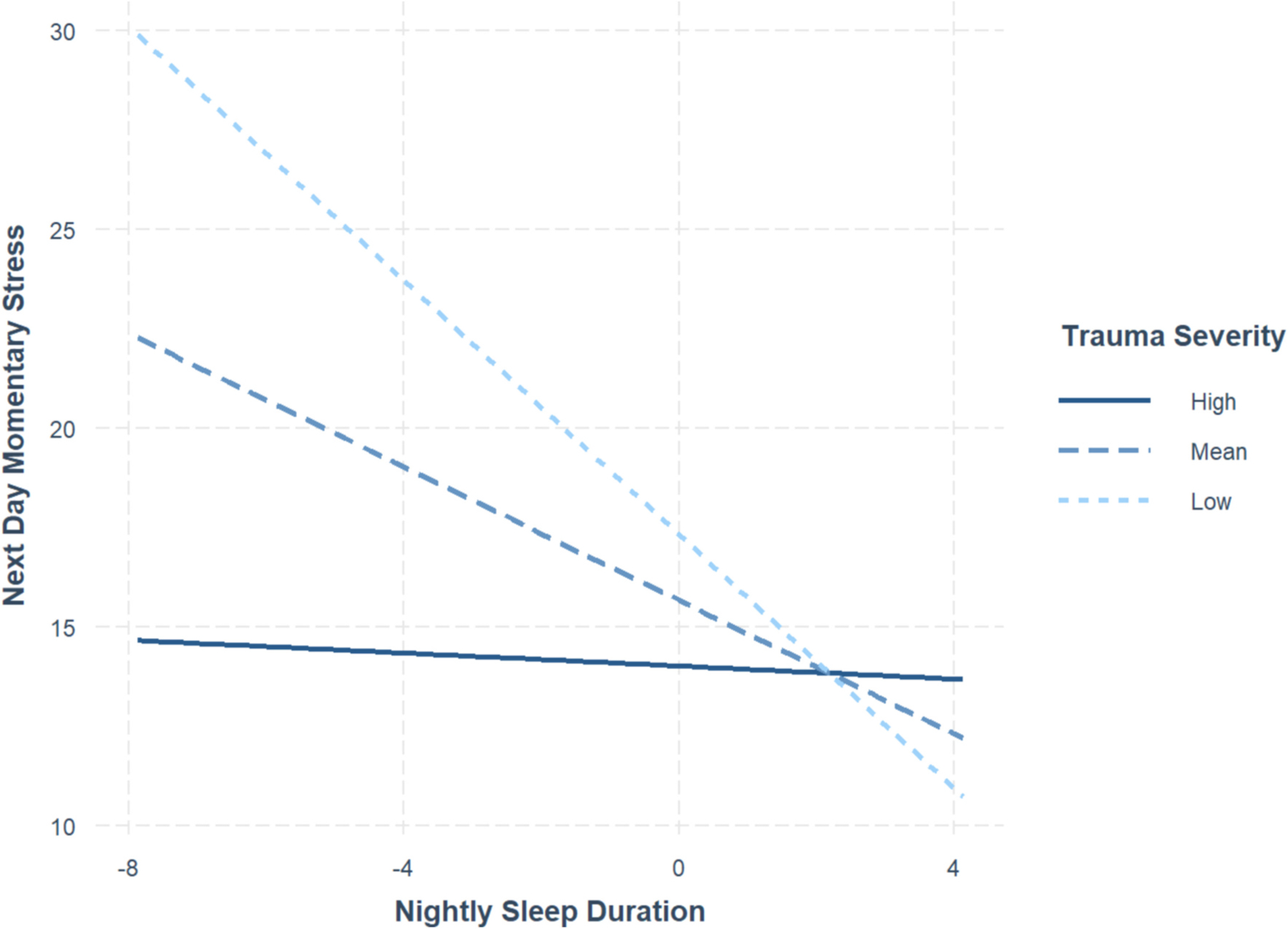
Trauma severity moderating association between sleep duration and momentary stress Note. Multilevel regression and simple slopes analyses show that trauma severity significantly moderated the negative association between sleep duration and momentary stress, such that the association is significant at lower levels of trauma severity but insignificant at lower levels. Because sleep duration was grand-mean-centered, its values range from −8 to 4 h.

Secondly, trauma severity significantly moderated the negative association between sleep duration and next-day momentary stress in the whole sample, regardless of group status. Simple slope analysis (see Fig. 4) revealed that the association was significant at one standard deviation below, β = −1.60, SE = 0.39, *p* < .001, and at the mean, β = −0.84, SE = 0.25, *p* < .001, trauma severity score but not significant at one standard deviation above the mean, β = −0.08, SE = 0.33, *p* = .81. In other words, the effect of nightly sleep duration on next-day momentary stress is significant and negative, but only at lower levels of trauma severity for the whole sample.

## 4. Discussion

The current study examined the dynamic associations between trauma severity, nightly sleep duration, and momentary perceived stress in a sample of FHR and non-psychiatric youth. Our findings suggest FHR youth experience higher trauma severity and shorter sleep duration than non-psychiatric youth, both of which were associated with higher next-day stress in the entire sample. Additionally, adolescents who reported lower trauma severity experienced less momentary stress on days following longer sleep duration, providing possible clues regarding the protective effects of sleep duration on experiences of everyday stress. Though findings should be taken with caution due to the small sample size, they may provide a nuanced perspective of the diathesis-stress conceptualization of sleep dysfunction and a foundation for future work focused on these dynamic associations in populations at-risk for psychopathology. Furthermore, this work could inform early intervention programs seeking to identify and target combinations of risk factors that may interact with genetic vulnerability and further contribute to symptomology and onset of psychosis later in life.

As hypothesized, the FHR group reported higher trauma severity and shorter sleep duration than the non-psychiatric group, consistent with previous work and across other psychosis-risk youth samples (Thompson et al., 2009; Addington et al., 2013; Brandt et al., 2023; Lunsford-Avery et al., 2015; Lunsford-Avery et al., 2017; Poe et al., 2017; Zaks et al., 2022). Youth at FHR for serious psychopathology may be at heightened risk of experiencing trauma due to genetic vulnerability and/or environmental proximity to affected, biological relatives (Brandt et al., 2022). Moreover, shortened sleep duration in the FHR group was unsurprising given sleep dysfunction is a common characteristic of prodromal psychosis (Yung and McGorry, 1996). Additional work is needed to expand the current work and assess if other trauma and sleep domains are present in FHR youth.

Contrary to predictions, FHR youth did not report higher overall stress in daily life compared to non-psychiatric peers. It was expected that momentary perceived stress would be higher in FHR adolescents because genetic/familial vulnerability is linked to psychosocial stress in the diathesis-stress model (Walker and Diforio, 1997), and numerous studies have found elevations in psychophysiological stress across the psychosis-spectrum (Carol and Mittal, 2015; Cullen et al., 2020; Brandt et al., 2023). However, it is possible that this null finding may reflect the presence of impaired emotional awareness (i.e., alexithymia) and regulation, documented neuropsychological phenomena in FHR and CHR individuals (Van’t Wout et al., 2007; Kimhy et al., 2015; van Rijn et al., 2011), such that the FHR participants may be less attuned to everyday affective experiences (e.g., psychological stressor) relative to controls. Alternatively, stress abnormalities may only emerge at later stages of psychosis progression, such as following the emergence of subsyndromal or prodromal symptoms in CHR (Georgiades et al., 2023; Millman et al., 2018; Muñoz-Samons et al., 2021) and full-blow psychosis (Pruessner et al., 2011; Zhou et al., 2024; Mondelli et al., 2010). Future work should leverage EMA and its high ecological validity to examine temporal and contextual factors on everyday stress indices beyond nightly sleep and in individuals across the psychosis-spectrum. Further, the impact of alexithymia on affective EMA data should be addressed in order to accurately assess daily emotional experiences in at-risk and general populations.

In the entire sample, those who reported more traumatic experiences and shorter sleep duration tended to experience higher levels of stress in daily life. Findings add to previous work suggesting trauma exposure and sleep dysfunction are related to heightened psychophysiological stress (Baddam et al., 2019; Lardinois et al., 2011). These results may provide support for the diathesis-stress conceptualization (Lunsford-Avery and Mittal, 2013) asserting the effect of environmental insults (e. g., trauma) and sleep dysfunction on psychological stress – although additional work is needed to replicate the findings. Furthermore, findings also align with evidence of heightened stress activity in samples diagnosed with trauma-related (Hu et al., 2014; Young and Breslau, 2004) and sleep-related disorders (Garefelt et al., 2020; Hansen et al., 2021). Subsequent work can explore factors that may drive the associations or compromise the individual variables in at-risk populations, such as retrospection or alexithymia (Alimoradi et al., 2022; Cai et al., 2023; Feyzioğlu et al., 2023).

Although momentary stress did not differ across levels of trauma severity in FHR youth, non-psychiatric adolescents who reported more severe trauma tended to experience higher momentary stress in daily life. These results complement research on general adolescent development suggesting that trauma can impact experiences of stress in all adolescents, regardless of psychosis-risk or underlying psychopathology (Costello et al., 2002; Baddam et al., 2019; Lardinois et al., 2011). Although null findings should be interpreted with caution, findings of the noted associations in the non-psychiatric group but not the FHR group may hint at the emergence of stress habituation, the process by which one’s biological stress response decreases with repeated stress exposure (Grissom and Bhatnagar, 2009), in these at-risk adolescents. Perhaps, growing up with a family member with psychosis is a lifelong, persistent stressor that desensitizes an adolescent to later stressors (Collip et al., 2011; Habets et al., 2012). If so, it may be reasonable that stress habituation serves as a coping mechanism to protect against repeated/subsequent stressors and accompanying distress. To further understand this possible interpretation and make definitive conclusions, additional research is needed with a more comprehensive battery of stress measures and larger samples.

In the whole sample, adolescents with less severe trauma displayed lower momentary stress on days following longer sleep duration. Sleep duration had no impact on next-day stress in youth with more severe trauma. Previous work has shown that longer sleep duration typically buffers for elevations in next-day stress in general populations (Yap et al., 2020), suggesting that the association between longer sleep duration and lower next-day stress may represent a healthy underlying link between sleep and stress processes. As such, our findings may reflect the long-term health benefits of having minimal exposure to early life trauma, such that the sleep-stress link is maintained in youth with low exposure to severe trauma but disrupted in those with substantial exposure to severe trauma. This is a point for further inquiry.

Despite the strengths of the study (e.g., relatively strong ecological validity, repeated measures with EMA, and longitudinal study design), there are important limitations that point to opportunities for future research. First, despite the advantages of using EMA and MLM, our statistical power was likely impacted by the small sample size, attrition, and low response rates. These limitations are common among psychosis research, especially when recruiting from at-risk youth populations and using longitudinal and EMA designs (Zammit et al., 2013; Moitra et al., 2017; Harvey et al., 2021). To maximize power, we did not exclude participants with missing data at six-month follow-ups nor with low response rates (<30 %); nonetheless, we recommend that future work prioritize recruiting larger, more diverse, at-risk samples with additional efforts to improve sample retention and adherence. Second, EMA data was only collected on weekends but not during the school week. Adolescence is marked by the balance of academic responsibilities, extracurricular activities, first-time employment, and peer networks. Each factor can serve as a potential psychosocial stressor and produce fluctuations in everyday sleep- and stress-related experiences. Thus, only collecting EMA data across three consecutive weekends does not account for weekday-specific fluctuations, thereby compromising our findings’ generalizability to weekday sleep and stress processes. Third, we used self-reported sleep duration, a measure vulnerable to recall bias, as our primary sleep parameter. Buysse (2014)’s RU-SATED model provides a multidimensional approach to conceptualizing sleep health, with sleep duration being a single facet of a complex system of sleep behaviors. Other important sleep dimensions (e.g., sleep quality, efficiency, timing, daytime naps and fatigue) were not represented in this study, such that we were unable to construct a comprehensive and ecologically rich profile of sleep health in FHR adolescents. Similarly, we did not differentiate between specific types of traumas (e.g., sexual abuse, physical neglect). Future work can address both limitations by exploring various sleep and trauma domains in order to capture nuanced sleep behaviors and traumatic experiences in at-risk youth.

These findings have important clinical implications. The broader literature presents various therapeutic approaches for targeting trauma, sleep difficulties, and maladaptive stress in general populations and across the psychosis-spectrum. For instance, trauma-focused cognitive behavioral therapy (CBT) appears to be a valuable intervention for PTSD in early life and adulthood (Kar, 2011; Mannarino et al., 2012; Arellano et al., 2014), with recent work supporting specialized applications for psychotic disorders and various traumas (Hardy et al., 2022). Similarly, CBT for insomnia (CBT–I) has been effective in mitigating PTSD symptoms and sleep problems in trauma-exposed individuals (Ho et al., 2016) as well as improving sleep and clinical outcomes across the psychosis-spectrum (Waite et al., 2023; Waters et al., 2020; Khalid, 2022; Bradley et al., 2018). Therapeutic use of melatonin also appears beneficial for patients with schizophrenia (Duan et al., 2021). Mindfulness-based intervention (MBI) is another useful approach for improving stress abnormalities, positive symptoms, and other clinical outcomes in people with schizophrenia (Hodann-Caudevilla et al., 2020; Jansen et al., 2020; Kim et al., 2021; Liu et al., 2021); however, this method has not been explored in a psychosis-risk sample. In all, CBT, MBI, and related interventions show great promise in addressing trauma, sleep dysfunction, and maladaptive stress at early stages of psychosis-risk and general populations – perhaps through promoting healthy sleep behaviors, coping strategies for trauma, and adaptive emotion regulation in everyday life.

## 5. Conclusions

Our findings contribute to the broader psychosis-risk literature suggesting that adolescents at-risk for psychosis experience more severe trauma and sleep dysfunction. We offer a novel perspective of the day-to-day impact of these problematic experiences on next-day psychological stress in FHR adolescents and non-psychiatric peers. Furthermore, those with less severe trauma tended to experience less stress on days following longer sleep duration, providing possible clues regarding healthy mechanisms underlying sleep duration and protective effects on stress-related experiences. With this, these findings provide a working framework for future research seeking to examine these patterns in both adolescents as a whole and adolescents at-risk for psychopathology.

## Supplementary Material

Supplement

## Figures and Tables

**Table 1:** Descriptive statistics of demographics, symptoms, and study variables.

Variables	Non-Psychiatric (*n* = 19)	Familial High-Risk (n = 19)	Total (*n* = 38)	Statistic
Age, mean (SD)	16.16 (1.8)	16.32 (2.1)	16.24 (1.9)	t(36) = −0.25, *p* = .80
Sex, *n* (%)				χ^2^(1) = 0.12, *p* = .73
Male	6 (31.6 %)	7 (36.8 %)	13 (34.2 %)	
Female	13 (68.4 %)	12 (63.2 %)	25 (65.8 %)	
Race, *n* (%)	47.4 %	63.2 %	55.3 %	χ^2^(1) = 0.96, *p* = .33
White	10 (52.6 %)	7 (36.8 %)	17 (44.7 %)	
Non-white	9 (47.4 %)	12 (63.2 %)	21 (55.3 %)	
SES, mean (SD)	37.87 (15.5)	33.70 (10.0)	36.2 (13.5)	t(23) = 0.82, *p* = .42
Symptoms, mean (SD)				
Positive	0.21 (0.4)	5.82 (2.1)	3.02 (1.0)	t(35.80) = −0.5.64, *p* < .001***
Negative	0.39 (0.6)	4.76 (2.9)	2.58 (1.5)	t(38.75) = −0.5.99, *p* < .001***
Disorganized	0.03 (0.1)	2.81 (2.0)	1.42 (0.8)	t(36.33) = −0.6.09, *p* < .001***
General	0.39 (0.6)	4.04 (2.1)	2.22 (1.2)	t(46.03) = −0.5.38, *p* < .001***
Study variables, mean (SD)				
Trauma severity	4.22 (3.7)	9.61 (8.9)	6.91 (7.2)	t(23.91) = −0.2.44, *p* = .02*
Sleep duration	8.06 (1.0)	7.30 (1.6)	7.68 (1.4)	t(29,76) = 1.78, *p* = .09
Momentary stress	13.04 (9.9)	18.52 (15.3)	15.78 (13.0)	t(30.87) = −1.31, *p* = .20
Attrition Rate, *n (%)*				
BL to 6 M	2 (10.5 %)	6 (47.4 %)	11 (28.9 %)	χ^2^(1) = 4.61, *p* = .03*
BL to 12 M	9 (31.6 %)	11 (57.9 %)	17 (44.7 %)	χ^2^(1) = 1.70, *p* = .19
Response Rate, mean (%, SD)	84.6 % (38.09, 9.8)	70.0 % (31.50, 12.9)	78.5 % (35.32, 11.6)	t(58.69) = 2.50, *p* = .02*

Note.

* *p* < .05

** *p* < .01

*** *p* < .001

All study variables (trauma severity, sleep duration, and momentary stress) and psychosis-risk symptoms are averages of within-person means. SES=Socioeconomic Status as categorized by Hollingshead (1975), SES was based on reported parental income and mother’s education level (father’s education level was not collected). For SES, there were *n* = 10 familial high-risk and *n* = 15 healthy controls included in the independent t-test. Attenuated psychotic symptoms were assessed using the Structured Interview for Prodromal Symptoms (SIPS; Miller et al., 2002; Miller et al., 2003). BL = baseline, 6 M = 6-month follow-up, 12 M = 12-month follow-up.

**Table 2:** Trauma and sleep predicting momentary stress in the whole group.

	Predictor: Sleep duration			Predictor: Trauma severity		
Predictors	Estimate	*SE*	*p*	Estimate	*SE*	*p*
(Intercept)	15.35	2.17	<0.001***	15.29	1.99	<0.001***
Between-person differences	−0.69	0.25	<0.01**	0.25	0.12	0.03*
Between-person averages	−0.18	1.63	0.91	0.30	0.30	0.33
Random effects						
Residual (σ^2^)	16.96			17.15		
Intercept (τ_00_)	12.91			11.99		

Note.

* *p* < .05

** *p* < .01

*** *p* < .001

Continuous Level-2 predictors (e.g., sleep duration and trauma severity) were grand-mean-centered. Between-person averages from the grand-mean-centered predictors were included as covariates.

**Table 3:** Associations between group status, sleep duration, trauma severity predicting momentary stress.

	Predictor: Sleep duration			Predictor: Trauma severity		
Predictors	Estimate	*SE*	*p*	Estimate	*SE*	*p*
(Intercept)	12.47	3.00	<0.001***	15.13	2.92	<0.001***
Between-person differences	−0.42	0.32	0.19	0.61	0.17	<0.001***
Between-person averages	0.75	1.69	0.66	0.47	0.34	0.18
Group status	5.88	4.42	0.19	2.02	3.37	0.65
Between-person differences × Group Status	−0.74	0.52	0.15	−0.71	0.23	<0.01**
Random effects						
Residual (σ^2^)	16.95			17.12		
Intercept (τ_00_)	12.65			12.21		

Note.

* *p* < .05

** *p* < .01

*** *p* < .001

Continuous Level-2 predictors (e.g., sleep duration and trauma severity) were grand-mean-centered. These multilevel moderations models examined how group status moderated the associations with between-person differences in trauma severity/sleep duration predicting momentary stress. Between-person averages from the grand-mean-centered predictors were included as covariates.

**Table 4:** Trauma and sleep interaction in predicting momentary stress in the whole sample.

	Outcome: Momentary stress		
Predictors	Estimate	*SE*	*p*
(Intercept)	15.28	2.04	<0.001***
Sleep duration			
Between-person differences	−0.91	0.27	<0.001***
Between-person averages	−0.28	1.53	0.85
Trauma severity			
Between-person differences	−0.23	0.20	0.25
Between-person averages	0.90	0.34	0.01*
Sleep Duration × Trauma Severity	0.10	0.04	<0.01**
Random effects			
Residual (σ^2^)	17.03		
Intercept (τ_00_)	12.86		

Note.

* *p* < .05

** *p* < .01

*** *p *< .001

Continuous Level-2 predictors (e.g., sleep duration and trauma severity) were grand-mean-centered. This multilevel moderation model examined the interaction of between-person differences in trauma severity and sleep duration when predicting momentary stress. Between-person averages from the grand-mean-centered predictors were included as covariates. Interaction between sleep duration and trauma severity is specifically between the between-person differences in sleep duration and trauma severity.
