# Supplementary material for: The day-to-day influence of trauma exposure and sleep dysfunction on everyday stress in youth at familial high-risk for psychotic disorders
Source: Schizophr Res. Author manuscript; Available in PMC 2025 Mar 1. (PMC11803678; doi:10.1016/j.schres.2024.10.024)
Supplement: Supplement [file NIHMS2045638-supplement-Supplement.docx]

***Supplementary Materials***

**Supplementary Analyses**

| **Table 1.** Bivariate Correlational Analyses | | | |
| --- | --- | --- | --- |
|  | Trauma severity | Sleep duration | Momentary stress |
| Age | 0.29 | -0.09 | 0.09*** |
| Sex | 0.08 | -0.05 | -0.09*** |
| Race | -0.10 | -0.15** | 0.00 |
| SES | -0.2 | -.02 | 0.08*** |

*Note.* *p*<.05*, *p*<.01**, *p*<.001***. Race was a binary coded variable with minoritized individuals coded as 1 and White/Caucasian participants coded as 0.

*Trauma Severity and Familial High-risk Status Predicting Nightly Sleep Duration*

Multilevel regression analyses reveal that there is no significant association between trauma severity and nightly sleep duration, β=.02, SE=.05, *p*=.68. Further, Group status does not significantly moderate this association, β=-.05, SE=.06, *p*=.36.

*Moderation of Participant Age*

Multilevel models showed that participant age significantly moderated the positive association between trauma severity and momentary stress, β=.28, SE=.09, *p*<.01. Simple slopes analysis shows that this association was significant and positive in older participants, β=.88, SE=.23, *p*<.001, but insignificant in younger participants, β=-.13, SE=.17, *p*=.44. In context, older participants who report more traumatic adverse events tend to experience higher momentary stress compared to younger participants.

Participant age further moderated the negative association between sleep duration and momentary stress, β=.50, SE=.14, *p*<.001. Simple slopes analysis shows that this association is significant and negative in younger participants, β=-1.54, SE=.35, *p*<.001, but insignificant in older participants, β=.25, SE=.37, *p*=.50. In other words, younger participants who receive shorter sleep duration are more likely to report higher momentary stress compared to older participants.
